# Biliary atresia

**DOI:** 10.4103/0971-9261.43015

**Published:** 2008

**Authors:** C. K. Sinha, Mark Davenport

**Affiliations:** Department of Pediatric Surgery, King's College Hospital, Denmak Hill, London SE5 9RS UK

**Keywords:** Biliary atresia, surgical jaundice

## Abstract

Biliary atresia (BA) is a cholangiodestructive disease affecting biliary tract, which ultimately leads to cirrhosis, liver failure and death if not treated. The incidence is higher in Asian countries than in Europe. Up to 10% of cases have other congenital anomalies, such as polysplenia, asplenia, situs inversus, absence of inferior vena cava and pre-duodenal portal vein, for which we have coined the term Biliary Atresia Splenic Malformation (BASM) syndrome. For these infants the aetiology lies within the first trimester of gestation. For others affected with BA, aetiology is more obscure and perinatal destruction of fully-formed ducts perhaps by the action of hepatotropic viruses has been suggested. Whatever the cause, the lumen of the extrahepatic duct is obliterated at a variable level and this forms the basis for the commonest classification (Types I, II, III). All patients with BA present with varying degree of conjugated jaundice, pale non-pigmented stools and dark urine. Key diagnostic tests include ultrasonography, biochemical liver function tests, viral serology, and (in our centre) a percutaneous liver biopsy. In some centres, duodenal intubation and measurement of intralumenal bile is the norm. Currently BA is being managed in two stages. The first stage involves the Kasai operation, which essentially excises all extrahepatic biliary remnants leaving a transected portal plate, followed by biliary reconstruction using a Roux loop onto that plate as a portoenterostomy. If bile flow is not restored by Kasai procedure or life-threatening complications of cirrhosis ensue then consideration should be given to liver transplantation as a second stage. The outcome following the Kasai operation can be assessed in two ways: clearance of jaundice to normal values and the proportion who survive with their native liver. Clearance of jaundice (<2 mg/dL or <34 µmol/L) after Kasai has been reported to be around 60%, whereas five years survival with native liver ranges from 40% to 65%.

Biliary atresia (BA) is a cholangiodestructive disease affecting both the intra- and extra-hepatic biliary tract ultimately leading to cirrhosis, liver failure and death if not treated.[1] The incidence is higher in Japan and China (1 in 9,600) than in Europe and the UK (1 in 16,000).[2,3]

## AETIO-PATHOGENESIS

Whatever be the cause, the end result is invariably complete obliteration of the lumen of extrahepatic bile ducts and progressive cellular inflammation of the intra-hepatic ducts.[4,5]

In some infants, there is evidence to suggest that the process begins early in gestation. Thus antenatal ultrasonography allows detection of that sub-group of BA that show cystic changes within the extrahepatic ducts.[6] Furthermore, about 10% cases of all cases have other congenital anomalies. These anomalies are unusual but characteristic such as polysplenia, asplenia, situs inversus, absence of inferior vena cava and pre-duodenal portal vein, for which we have coined the term Biliary Atresia Splenic Malformation (BASM) syndrome.[7,8] In these infants there is a high incidence of first trimester maternal problems such as diabetes and it is a reasonable supposition that all of the constituent anomalies occur during the critical period of organogenesis within that period.

There are some histological similarities between the appearance of developing bile ducts at the porta hepatis at 12-14 weeks gestation and the appearance of the residual biliary ductules at the porta hepatis in BA patients, suggesting that even some cases of isolated BA the disease may be due to alterations in early bile duct development and failure of remodeling.[9]

Alternatively, BA may arise due to damage of a fully-developed biliary tract at some point in post-or at least perinatal life. Thus some studies report an association of various gastrointestinal viral infections with BA including reovirus type 3,[10–13] cytomegalovirus,[14,15] respiratory syncitial virus,[16] Epstein-Barr virus,[17] human papillomavirus[18] and rotavirus type A.[19] However, other studies using similar serological and isolation techniques have found no relationship.[20–22] The most common hepatotropic viruses causing disease in older children (such as hepatitis A, B and C) have not been related to BA.[23,24]

There are also various animal models that can mimic some of the pathological features of BA and which rely upon exposure of the newborn animal to viruses such as rotavirus and reovirus.[25–27]

Genetics may play a role in the pathogenesis of BA, perhaps in predisposing to the detrimental effects of hepatotropic viral infection. Although in most, this plays probably a fairly minor role as the usual evidence for a genetic background is missing. Familial cases of BA have been rarely reported[28–31] and discordance has been reported in monozygotic twins.[32–35]

## PATHOLOGY

The gross appearance of the extrahepatic biliary tract varies from an inflamed, hypertrophic occluded biliary tract to an atrophic remnant. Histologically, the liver has features of portal tract inflammation, with a small cell infiltrate, bile ductule plugging and proliferation.[36] Later on, bridging fibrosis and ultimately biliary cirrhosis occurs.

**Figure 1:**
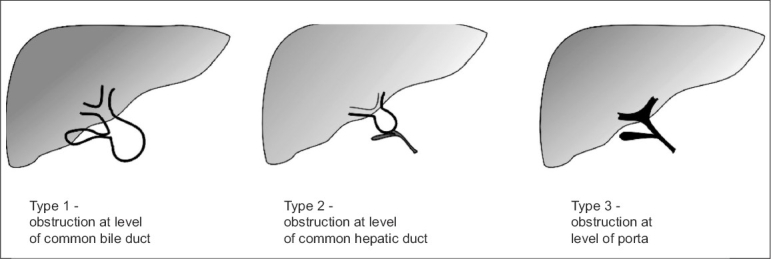
Types of Biliary Atresia

The lumen of the extrahepatic duct is obliterated at a variable level and this forms the basis for the commonest classification in clinical use - the Japanese Association of Pediatric Surgeons (JAPS) classification [Figure 1]. Type 3 is the commonest (∼90%) type and has the most proximal level of obstruction in the porta hepatis - no visible duct should be present. In type 2 BA, atresia extends up to common bile duct, whereas in type 1 BA, atresia extends up to common hepatic duct level.

An important variation is that of cystic change, seen in about 5% of cases, within some part of the extrahepatic biliary tract. Some cysts contain mucus, while others contain bile. If it is bile, there may be diagnostic confusion with that of a true choledochal cyst. In cystic biliary atresia, the wall is invariably thickened, lacks an epithelial lining and communicates poorly with abnormal non-dilated intrahepatic ducts. This should be evident at operative or percutaneous cholangiography.

## CLINICAL FEATURES

All patients with BA present with varying degree of jaundice, clay-colored stools and dark yellow urine. The severity of jaundice increases steadily and it is not unusual to find bilirubin levels around 20 mg% at the time of first presentation in developing countries. Failure to thrive, coagulopathy and anemia are also not uncommon. Some will present with signs of advanced disease and cirrhosis such as ascites, umbilical hernia, prominent abdominal veins and respiratory discomfort. In comparison to European or North American experience, most cases in developing countries present late. In a review of BA, only 5% cases were seen below 60 days of age, 40% between two and three months, 30% between three and four months and 25% presented beyond four months of age. Hepatomegaly was seen in all and 60% patients had a palpable spleen. *(Prof. DK Gupta personnel communication, AIIMS, New Delhi, India)*.

## DIAGNOSIS

The clinical diagnosis of BA is usually all too obvious in late-presenting cases. However, in infants of <60 days the diagnosis can be difficult. Key investigations include ultrasonography, biochemical liver function tests, viral serology, and a percutaneous liver biopsy. In some centers, duodenal intubation and measurement of intralumenal bile is the routine test for BA. Newer modalities such as ERCP[37] and MRCP have been used at times, although the former is clearly highly operator-dependent and the latter not sufficiently precise in its delineation of infantile biliary anatomy to offer real advantage. Possibly, in most surgical centers, operative cholangiography remains the principal investigation in demonstrating biliary patency. This can also be performed laparoscopicaly with apparently good results.[38]

**Liver function tests (LFTs):** LFTs are abnormal in all patients of BA. There is a rise in total serum bilirubin (mainly conjugated) and fall in serum proteins (especially albumin) and a reversal of the albumin/globulin ratio in advanced cases. Alkaline phosphatase and transaminase (e.g. sGOT, sGPT) levels rise. Deranged LFTs correspond to the degree of parenchymal damage rather than the duration of disease.

**Imaging:** Hepatobiliary ultrasound after 12 h of fasting (with intravenous fluid support) is perhaps the initial investigation of choice. Certainly BA is highly unlikely if a dilated intrahepatic biliary duct is found (being more indicative of an obstructed choledochal cyst, or inspissated bile syndrome). Percutaneous liver biopsy after exclusion of medical causes of cholestatic jaundice (e.g. alpha 1-antitrypsin deficiency, Alagille's syndrome, and neonatal hepatitis) is a helpful investigation in diagnosis, but relies upon expert pathological interpretation. Negative ultrasonography and positive histology results should be able to establish the correct preoperative diagnosis in about 85% cases of BA (39). ERCP may be considered in infants with equivocal biopsy results, although it should be noted that this diagnosis depends crucially on failure to show a biliary tree, and hence appropriate experience and judgment are essential.[37,39] Percutaneous transhepatic cholangiography (PTC) is valuable in those diagnostic groups in which dilated intrahepatic bile ducts are a feature. This is particularly so in the inspissated bile syndrome group, when not only can the diagnosis be established but an attempt at therapeutic saline lavage also can be made. About half of this group can be treated effectively without the need for laparotomy and without any recurrence or long-term sequelae.[39]

**Nuclear scanning:** Excretion of Technetium-labeled isotopes into the gut within 24 h, establishes the patency of the biliary tract. However, a negative scan even after 24 h may still be consistent with both BA and an advanced stage of neonatal hepatitis. Negative HIDA scans may be repeated after one week of phenobarbitone therapy, if clinically indicated. Phenobarbitone is known to stimulate the hepatic enzymes and increase the flow of bile. One study showed that 40% patients with neonatal hepatitis excreted the isotope into the gut within 24 h in post-luminal HIDA scan while that of BA again failed to do so. Alternatively, further improvements have been suggested with the addition of betamethasone to phenobarbitone prior to HIDA scanning.[40]

**Percutaneous needle biopsy:** Sometimes the interpretation of a biopsy can be difficult and needs an experienced pathologist as there is a lot of overlap in the histological finding of BA and neonatal hepatitis. There is also an inherent risk of bleeding in performing needle biopsy.

**Duodenal drainage test:** A four-hourly duodenal aspiration is done to confirm the presence of bile in it. Again, the test may be falsely negative in patients with severe neonatal hepatitis.

**Per-operative cholangiogram:** In most centers, having excluded medical causes of jaundice and failed to show isotope excretion in a HIDA scan, then progression to peroperative cholangiogram is a reasonable option. The key observation at laparotomy is presence or absence of bile in the gallbladder. Clearly in the presence of bile, apart from the rare type 1 cases, then BA can be excluded. A cholangiogram through the gallbladder should demonstrate the entire biliary tree, but in those cases, where only the distal common bile duct (CBD) opacifies, an attempt should be made to delineate the proximal intrahepatic tree by application of a distal vascular clamp.

## MANAGEMENT

Currently BA is being managed in two phases:

- *First phase*: An attempt to preserve the infant's own liver. This usually involves the Kasai operation which essentially excises all extrahepatic biliary remnants leaving a transected portal plate, followed by biliary reconstruction using a Roux loop onto that plate as a portoenterostomy.
- *Second phase:* If bile flow is not restored by Kasai procedure or life-threatening complications of cirrhosis ensue then consideration should be given to liver transplantation. Sometimes this is done as a primary procedure, in those who present late with features of advanced cirrhosis.

## SURGICAL TECHNIQUE

The major breakthrough in the surgery for biliary atresia was seen in 1959 when Morio Kasai reported the operative relief of biliary obstruction in infants traditionally considered to have non-correctable biliary atresia.[41] As described by Kasai and modified subsequently by others, the surgical steps of portoenterostomy starts with opening of the abdomen through a right upper transverse incision, a peroperative cholangiogram (if required) and a wedge liver biopsy (if needed). Once the diagnosis of BA is established the liver should be mobilized fully outside of the abdominal cavity (by division of its suspensory ligaments), to ensure maximal exposure of the porta hepatis and facilitate a detailed dissection. The atretic gall bladder, cystic duct and all the remnant extrahepatic biliary tree are excised up to the level of the porta hepatis. The portal dissection itself must be wide extending from exposure of the origin of the umbilical vein from the left portal vein (in the Rex fossa) to the bifurcation of the right portal vein pedicle. Small veins from the portal vein to the portal remnant should also be divided to expose the caudate lobe posteriorly. The correct level of transaction is flush with the liver capsule, where the plane is usually self-evident. Transgression into actual liver parenchyma, however, adds nothing to the success of surgery. Most transected ductules are found in the marginal areas, recapitulating the normal biliary arrangement, and it is important to allow these to drain into a long (∼40 cms) Roux loop of jejunum. Modifications such as an intussusception valve, stomas or implanting the distal end of the Roux into the duodenum have no real advantages in clinical practice and have been discontinued in most centers.[42]

Various technical variants have been proposed according to the anatomical pattern of the biliary remnant. Hepaticojejunostomy may be possible in Type 1 BA although a complete excision and portoenterostomy even in these is probably a better option. If the gallbladder and distal common bile duct are patent then anastomosis of the opened gallbladder to the conduit to the transected portal plate (porto-cholecystostomy) is possible and avoids post-operative cholangitits.[43] The appendix has also been described as a conduit (appendicojejunostomy),[44] although some later series suggests no real advantages.[45]

A number of drugs have been suggested to try and improve postoperative results. For instance, there are anecdotal and uncontrolled studies suggesting benefit from corticosteroids,[46,47] ursodeoxycholic acid[47] and even Chinese herbs.[48] However, none have been subjected to anything like acceptable scientific scrutiny. The initial results of a randomized double-blind, placebo controlled trial using post-operative prednisolone (2 mg/kg/day) showed a reduction in initial bilirubin levels but no significant difference in ultimate clearance of jaundice or reduction in need for transplantion.[49]

## COMPLICATIONS

Early postoperative complications include: cholangitis, bleeding, leak from anastomosis, prolonged ileus, and intestinal obstruction. Late complications include: cessation of bile flow, recurrent cholangitis, portal hypertension, ascites, hepato-pulmonary syndrome, and formation of bile lakes in the liver and cirrhosis.

### Cholangitis

Cholangitis occurs in 30–60% of cases in the first two years following the Kasai procedure.[50–52] The severity can vary from mild to fulminant sepsis. Clinically, the patient will develop fever or hypothermia, vomiting, jaundice, hepatosplenomegaly, abdominal pain/distension and acholic stools. The diagnosis can be confirmed by blood culture (and conduit cultures if present) and/or liver biopsy.[50] However, although Gram-negative organisms or a mixed flora can be seen, mostly cultures are negative. The cause of cholangitis is not clear but there must be an intestinal-biliary communication and therefore the most favored hypothesis is that of an ascending infection from the gut.

Treatment includes resuscitation, intravenous fluid, broad spectrum antibiotics, (and in some centers high doses of steroids) for 7–10 days. In recurrent or late-onset cholangitis, obstruction to the drainage of the Roux loop should be considered.[53] In those where a mechanical cause can not be found, a prolonged course of antibiotics should be considered.

Historically, various measures have been tried with to reduce cholangitis although none have stood the test of time. These include stomas or catheters in the proximal limb of the Roux loop,[54,55] use of an omental wrap applied to the porta hepatis,[56] jejunal loop "valves",[57] creation of an intussuscepted ileocecal conduit[58] and use of an appendiceal conduit based on its vascular pedicle.[44] Roux loop stomas are associated with problems such as prolapse, retraction, fluid and electrolyte loss, bleeding and malabsorption and have been largely abandoned.

## PORTAL HYPERTENSION

The incidence of portal hypertension is about 75% after Kasai operation[59,60] and has a clear relationship with liver fibrosis. In a recent study, it was found that increased portal pressure (measured at the time of Kasai operation) is a bad prognostic sign.[61] These patients will have higher chances of developing portal hypertension, even if bilirubin level normalizes after operation. This study reported that portal pressure index (height of the saline level column above the liver surface level) was safe, simple and better predictor of postoperative outcome than a hepatic fibrosis score.[61] Portal hypertension may cause clinically significant variceal formation at the oesophagus, stomach, Roux loop and/or rectum. In patients with good liver function, varices are treated with endoscopic sclerotherapy or banding;[62] but in those with persisting jaundice, poor synthetic liver function, this will only be temporizing measure and liver transplantation is the only really successful option. Interventional radiological techniques such as transjugular intrahepatic portosystemic shunts (TIPS) are possible for some cases, as a bridge to transplantation, but require skill and perseverance if good results are to be obtained. Portal vein hypoplasia may even preclude this option. In those unusual cases of life-threatening bleeding, but non-progressive liver disease and good liver function, a conventional portosystemic shunts should be considered, particularly if transplantation is not an option.

## HEPATO-PULMONARY SYNDROME AND PULMONARY HYPERTENSION

Hepato-pulmonary (HP) syndrome is characterized by hypoxia, cyanosis, dyspnoea and clubbing and is due to development of pulmonary arterio-venous shunts. This seems to be due to gut-derived vasoactive substances that are not cleared by the cirrhotic liver. The diagnosis is made by pulmonary scintigraphy. HP syndrome can be reversed after liver transplantation.[62,63]

Pulmonary hypertension can also be a late complication of the cirrhotic liver[64] and can be diagnosed by echocardiography. In some, liver transplantation may be indicated.

## BILIARY LAKES

Bile containing cysts can develop in the liver postoperatively even in patients with complete clearance of jaundice. If infection and cholangitis supervene then such bile lakes can be drained either externally or internally by cyst-enterostomy. Ultimately, liver transplantation may be required as this usually implies marked degenerative cirrhotic change.

## MALIGNANCIES

A number of malignancies have been reported in the cirrhotic livers of patient with biliary atresia including hepatocellular carcinoma,[65] hepatoblastoma[65] and cholangiocarcinoma.[66]

## OUTCOME

The outcome following the Kasai operation can be assessed in two ways:

- Clearance of jaundice
- Proportions of native liver survival

Clearance of jaundice (<2 mg/dL or <34 *µ*mol/L) after Kasai has been reported to be around 60%,[67,68] whereas five-year survival with native liver ranges from 35% to 64%.[2,69–71] Longterm (10 years or greater) survival have been reported in the range of 27%-53%.[2,71,72] In our series, only about 15% had true longterm survival without jaundice, normal liver biochemistry and no signs of liver disease or portal hypertension.[72] However, even in this apparently "cured" group, liver histology still remains abnormal.[72]

In the children with a failed Kasai operation, liver transplantation is the only hope. The main problems associated with this major operation are lack of a suitable donor, small size of the recipient abdomen, immunosuppression and a significant postoperative morbidity and even mortality. At present, three-year survival after liver transplantation in good centers is 85–90%.[73,74]

## PROGNOSTIC FACTORS

Biliary atresia is a rare disease and surgical outcome following biliary atresia depends upon adequate dissection and restoration of bile flow, together with effective treatment of the two major complications (cholangitis and portal hypertension). A number of factors contribute to good outcome and may be listed as:

- Operator experience and size and experience of referral centre.
- Extent of the pre-existing liver damage
- Frequency of cholangitis
- Syndromic (e.g. BASM) patients have poorer prognosis than non-syndromic group. This may be due to severe cardiac disease and hepatopulmonary syndrome.[7,8]
- Age at operation- The age at Kasai operation has an effect on outcome, but it is not as clear-cut as was once thought. There is no real cut-off (e.g. six or eight weeks or 60 days). These were simply the arbitrary values from earlier small studies. There is no doubt that the fibrotic element of the hepatopathology is progressive but beyond what age an attempt at a Kasai portoenterostomy is pointless, is not known. Some infants will come to surgery early with very soft, non-fibrotic livers, at 20 days for instance, but because they have few residual ductules at the porta hepatis their Kasai's will fail. In contrast, some infants will be operated late (beyond 100 days) and 10-year survival in these infants could be 40%.[75]

**Figure 2:**
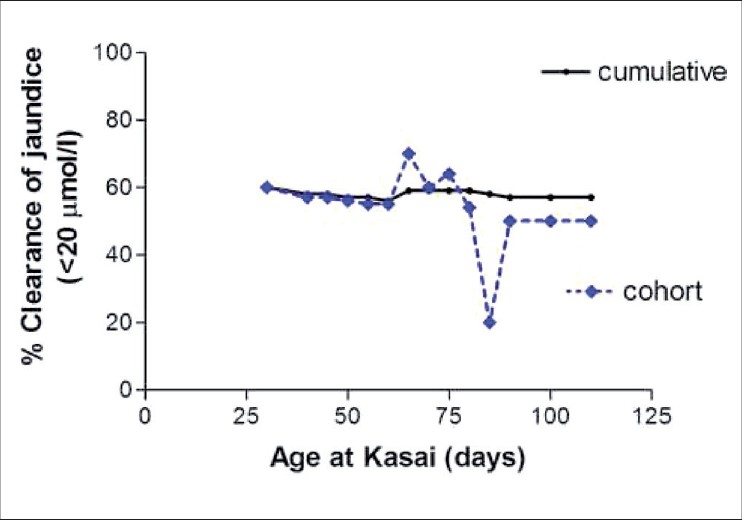
Clearance of jaundice (<20 µmol/L) by age cohort and cumulatively for Isolated. Biliary atresia (*n* = 177)

**Figure 3:**
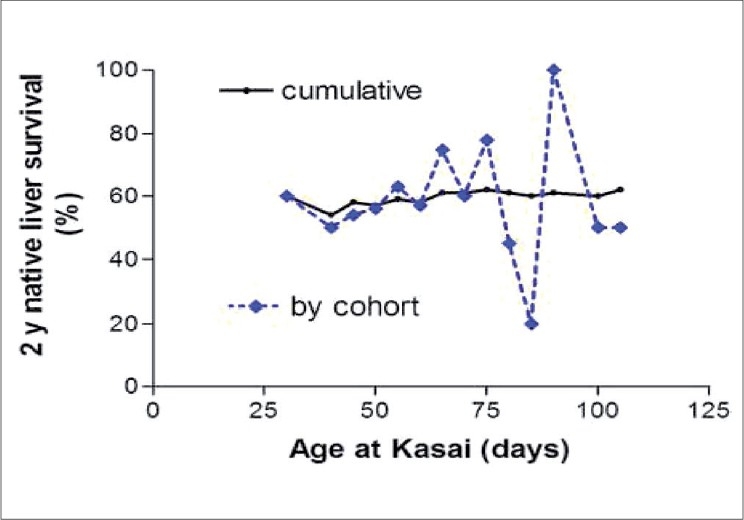
Two-year native liver survival by age cohort and cumulatively for Isolated. Biliary atresia (*n* = 177)

In a recent study at our center, an age cohort analysis was used to determine the effect of age at Kasai operation in 225 patients. Infants were divided into sequential cohorts by age at Kasai operation (e.g. ≤30, 31-40, 41-50 days etc.). The outcome was measured using clearance of jaundice to <20 *µ*mol/L and native liver survival at two years post-operation. In the Isolated BA group, the clearance of jaundice was seen in 56% [Figure 2] and 60% retained their native liver at two years [Figure 3]. There was no overall statistical difference in probability to clear their jaundice with age at surgery or probability of native liver survival with age at surgery. Conversely, in BA infants with a presumed developmental origin (i.e. BASM or Cystic Biliary Atresia), increasing age had a significant detrimental effect in outcome.
